# Evaluation of Novel Antibacterial *N*-Halamine Nanoparticles Prodrugs towards Susceptibility of *Escherichia coli* Induced by DksA Protein

**DOI:** 10.3390/molecules20047292

**Published:** 2015-04-21

**Authors:** Qigeqi Dong, Alideertu Dong, Morigen

**Affiliations:** 1 College of Life Science, Inner Mongolia University, Hohhot 010021, China; E-Mail: zhylthu@sina.cn; 2 College of Chemistry and Chemical Engineering, Inner Mongolia University, Hohhot 010021, China

**Keywords:** *N*-halamine, silica, polystyrene, nanoparticles, antibacterial, DksA protein, *E. coli*

## Abstract

Novel *N*-halamine nanoparticles potentially useful for killing pathogenic bacteria, *i.e.*, SiO_2_@PS/*N*-halamine NPs, were successfully synthesized via the immobilization of *N*-halamines onto the polystyrene-coated silica nanoparticles (SiO_2_@PS NPs). The effect of reaction conditions, *i.e.*, chlorination temperature, bleaching concentration, chlorination time, on the oxidative chlorine content in the products was systematically investigated. The antibacterial activity of the products was tested via the modified plate counting methd using *Escherichia coli* (*E. coli*) as a model bacterium. The possible mechanism of the antibacterial action of the products was also studied using scanning electron microscopy combined with a inhibition zone study. The antimicrobial capability of the products was well controlled by tuning the oxidative chlorine content in the products. More importantly, the role of DksA protein in the susceptibility of *E. coli* against the products was proven using a time-kill assay. This in-depth investigation of the sensitivity of *E. coli* towards *N*-halamine NPs provides a systematic understanding of the utility of *N*-halamines for deactivating bacteria or even disease control.

## 1. Introduction

Bacterial invasions are a major cause of presently increasing lethal diseases [1]. In response to increasing diseases induced by pathogenic bacteria, antibacterial prodrugs with capability of killing pathogenic bacteria are urgently required. *N*-Halamines are attracting considerable interest in this context due to their characteristics, *i.e.*, powerful antibacterial activity, duration of action, long-term stability, and regenerability [2]. *N*-Halamines with nitrogen-halogen covalent bonds in their structure are typical oxidative agents, and the halogen with “+1” oxidation state possesses a strong tendency to kill bacteria [3]. Their antimicrobial action is considered to be a manifestation of a chemical reaction involving the transfer of oxidative halogen from the *N*-halamines to the bacterial cells, followed by inhibition or even destruction of the batcteria’s enzymatic and/or metabolic cell processes, resulting in the death of the bacteria [4]. Consequently, *N*-halamines are applied for this purpose in a wide variety of fields, including water purification systems, food storage and packaging, medical devices, hospitals, hygienic products, dental office equipment, household sanitation, *etc.* [5]. *N*-Halamines can always be composed of one or more imide/amide/amine *N*-halamine bonds, and their stability is in the order amine > amide > imide [5]. 

Poor stability can seriously restrict the practical utilization of an *N*-halamines in many fields. Nanoparticles (NPs) show overwhelming superior reactivity compared to their bulk counterparts due to their smaller size and larger active surface area [6]. Thereby, developing *N*-halamine NPs could be an effective way of enhancing their biocidal activity. The colloidal template method is an effective and simple way of producing NPs with desirable structures and properties [7]. Inorganic carriers, e.g., silica NPs, have been widely employed to deliver antimicrobial molecules by grafting them on the surface or loading them into the pores of the NPs [8]. Accordingly, anchoring *N*-halamines onto silica NPs is a reasonable approach to improve their antimicrobial activities. However, it is difficult to immobilize *N*-halamines directly onto bare silica NPs merely using the hydroxyl groups on the silica surface [9]. To overcome this problem, a chemical linker between the silica NPs and *N*-halamines is needed. Polystyrene (PS) can be easily coated on the surface of silica NPs by a polymerization process, and chloromethylation can make the outer PS layers more reactive to facilitate the subsequent chemical binding with functional components [10]. In this work, *N*-halamines were successfully attached onto silica NPs using chloromethylated PS layers as linkers.

Despite the numerous advances that have been achieved, in-depth studies of the antibacterial action of *N*-halomines against bacterial pathogens are quite rare. Herein, *N*-halamine NPs were easily synthesized by immobilizing *N*-halsamines on PS-coated SiO_2_ NPs, and more importantly the susceptibility of bacteria towards the resulting *N*-halamine NPs was examined. Some bacterial proteins, e.g., the DksA protein in *Escherichia coli* (*E. coli*), have been proven to be involved in the cell damage repair mechanism for adaptive survival responses [11]. Cell damage would occur in absence of these proteins, which could result in a wide range of functional disturbances and even cell death if not restored in due course. In this study, the effect of DksA protein on the susceptibility of *E. coli* to antibacterial *N*-halamine NPs was investigated systematically. In light of such an in-depth study, it would be valuable to clarify the role of DksA, and thus extend the range of applications of *N*-halamine NPs.

## 2. Results and Discussion

### 2.1. Synthesis of SiO_2_@PS/N-halamine NPs

**Figure 1:**
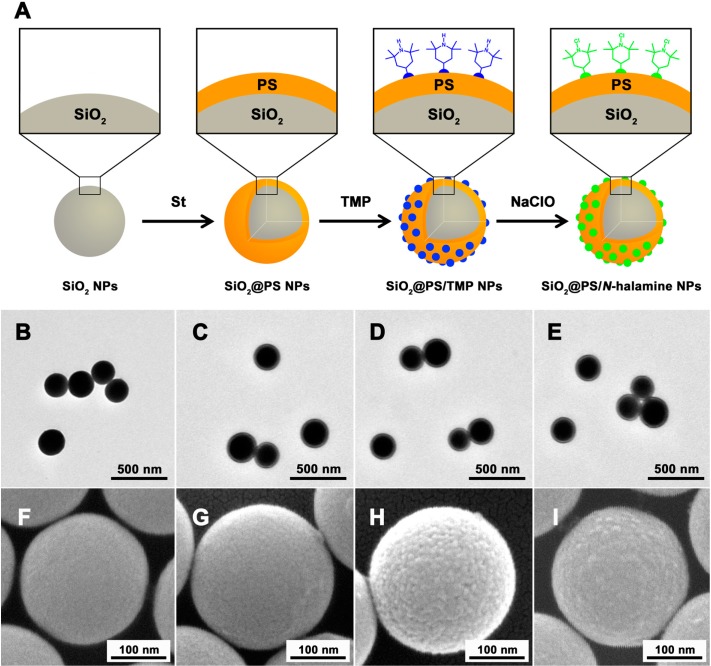
(**A**) Synthesis procedure of SiO_2_@PS/*N*-halamine NPs; (**B**–**E**) TEM and (**F**–**I**) SEM of SiO_2_ NPs (B and F), SiO_2_@PS NPs (C and G), SiO_2_@PS/TMP NPs (D and H), and SiO_2_@PS/*N*-halamine NPs (E and I).

The TEM and SEM techniques were applied to verify the morphology, shape, surface state, and size of the products. From the TEM image in Figure 1B, we can see that the SiO_2_ NPs are monodisperse, spherical, and smooth. In Figure 1C–E, the SiO_2_@PS NPs, SiO_2_@PS/TMP NPs, and SiO_2_@PS/*N*-halamine NPs show defined spherical shapes and visible core-shell structures, implying the *N*-halamine was grafted outside the PS-coated SiO_2_ NPs. The average diameter is 233.3 nm for SiO_2_ NPs, 259.4 nm for SiO_2_@PS NPs, 261.5 nm SiO_2_@PS/TMP NPs, and 268.4 nm for SiO_2_@PS/*N*-halamine NPs, respectively. The gradual increase in particle size is another confirmation of the formation of the SiO_2_@PS/*N*-halamine NPs. The TEM image also shows that all these NPs offer a quite narrow normal size distribution. The immobilization of *N*-halamine causes the distinction in the surfaces of NPs shown in the SEM images (Figure 1F–I). Quite smooth appearances were observed for both SiO_2_ NPs and SiO_2_@PS NPs, and no significant differences between them were found, except for the size change. Comparatively, the surface states changed significantly, and lots of dense small dots appeared after the *N*-halamine was anchored on the SiO_2_@PS NPs. Such rough surfaces for SiO_2_@PS/TMP NPs and SiO_2_@PS/*N*-halamine NPs suggest that SiO_2_@PS NPs were successfully modified with the *N*-halamine component.

**Figure 2:**
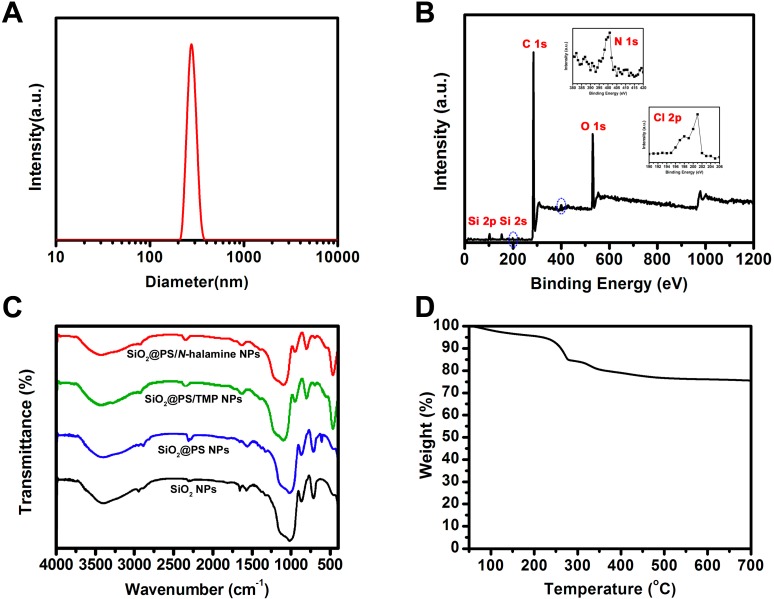
(**A**) DLS analysis and (**B**) XPS spectrum of SiO_2_@PS/*N*-halamine NPs; (**C**) FTIR spectrum of SiO_2_ NPs, SiO_2_@PS NPs, SiO_2_@PS/TMP NPs, and SiO_2_@PS/*N*-halamine NPs; (**D**) TGA result SiO_2_@PS/*N*-halamine NPs.

The size distribution of the SiO_2_@PS/*N*-halamine NPs was characterized by dynamic light scattering (DLS) as shown in Figure 2A. The DLS result illustrates that the NPs have sizes ranging from 220 nm to 380 nm, with an average size of about 283.4 nm. As expected, the sizes detected by DLS are always larger than those found by electron microscopy [12]. The most plausible reason is that the electron microscopy tests are carried out at a dry state, while swelling behavior occurs in the solvent medium during DLS characterization [13]. Besides, the shrinking of the sample for TEM and SEM tests caused by electron beam damage is also a possible explanation for the size differences seen between the electron microscopy and DLS results [14].

Detailed information about the composition of the SiO_2_@PS/*N*-halamine NPs was obtained by X-ray photoelectron spectroscopy (XPS) measurements. The XPS detection depth for materials is less than 10 nm, thus XPS analysis is always applied for the examination of the chemical information either at or very near a material’s surface [15]. The XPS spectrum of the SiO_2_@PS/*N*-halamine NPs is shown in Figure 2B. The characteristic peaks, assigned to photoelectrons originating from the Si 2s, Si 2p, and O 1s energy levels, appear at 154, 103, and 533 eV, respectively, and they act as unique elemental markers for the silica components [16]. Two elemental Si 2s and Si 2p signals are also likely due to the silicon wafer support used for sample immobilization. An intense peak at 285 eV, corresponding to the C 1s information, is mainly from the PS layer. The C 1s peak is always used for the calibration of all the binding energies [17]. More importantly, some relatively weak peaks, N 1s at 400 eV and Cl 2p at 200 eV, are also detected in the XPS spectrum, and are clearly seen in the magnified version in the insert of Figure 2B [18]. The apperance of these two peaks provides conclusive evidence for the presence of the *N*-halamine component.

The FTIR spectra of the products were recorded to verify the formation of the functional groups (Figure 2C). As for SiO_2_ NPs, the peak at about 960 cm^−1^ is attributed to the Si-OH stretching, and the two peaks at about 800 and 1090 cm^−1^ arise from the symmetric and antisymmetric stretching vibrations of the Si-O-Si bond, respectively [19]. The appearance of Si-OH and Si-O-Si peaks acts as marker for SiO_2_ NPs. Besides, the absorption band at about 1630 cm^−1^ is attributed to the residual water, and the peaks at about 1450 and 1400 cm^−1^ are ascribed to the unhydrolyzed SiOC_2_H_5_ and the symmetric bending vibration of the C-H bond, respectively. In the spectrum of SiO_2_@PS NPs, the characteristic absorption bands at about 1630, 1500, and 1450 cm^−1^ for C=C stretching and at about 750 cm^−1^ for C-H bending of the benzene ring are clearly observed. The peak at about 700 cm^−1^ is attributed to a corrugation vibration of the benzene ring. The peaks at about 2995 and 2926 cm^−1^ correspond to the C-H bond stretching vibration. These peaks confirm the existence of PS polymer. After anchoring TMP on SiO_2_@PS NPs, an obvious N-H peak appears at about 3430 cm^−1^, suggesting that the TMP component is immobilized successfully onto the surface of the SiO_2_@PS NPs. Upon the bleaching treatment, an almost invisible N-H stretching vibration at about 3430 cm^−1^ is associated with the N-H→N-Cl transformation [20]. This negligible weak N-H peak possibly corresponds to the unchlorinated amine groups.

Thermogravimetric analysis (TGA) was carried out to quantify the composition and content information of each component in the SiO_2_@PS/*N*-halamine NPs as shown in Figure 2D. There are mainly two characteristic weight loss regions, which imply that there are three different substances in the NPs. The weight loss below 200 °C is attributed to the evaporation of water and residual organic solvent [21]. The NPs begin decomposing at about 200 °C, which can be ascribed to the decomposition of the *N*-halamines. The decomposition rate increases when the temperature reaches about 300 °C, which is caused by degradation of the polystyrene layer. The weight completely disappears at about 700 °C and only silica resides are left behind [22]. From the TGA result, we can estimate that the *N*-halamine content is about 12.8 wt %.

The formation of the *N*-halamines was further substantiated via a modified iodometric/thiosulfate test [23]. In the iodometric/thiosulfate test color changes are evidence of oxidation-reduction reactions. The oxidative chlorine in the SiO_2_@PS/*N*-halamine NPs suspension firstly oxidizes iodide ions to produce iodine in acidic condition, which leads to a chromogenic reaction in starch solution to show a blue color. The iodine is then titrated using thiosulfate to return back to a quasi-transparent solution. Finally, the *N*-halamines are recovered after the simple bleach treatment [24]. These color changes prove well the presence of oxidative chlorine, reflecting as a result the presence of *N*-halamines in the NPs.

### 2.2. Effect of Reaction Conditions on Oxidative Chlorine Content

*N*-halamines are always prepared by halogenating their corresponding imide/amide/amine precursor [25]. Therefore, the reaction conditions of the chlorination process are quite important for controlling the loading of the oxidative chlorine atoms on the *N*-halamines. In this study, the effect of chlorination conditions, *i.e.*, reaction temperature, bleaching concentration, chlorination time, on the oxidative chlorine content in the SiO_2_@PS/*N*-halamine NPs was investigated.

**Figure 3:**
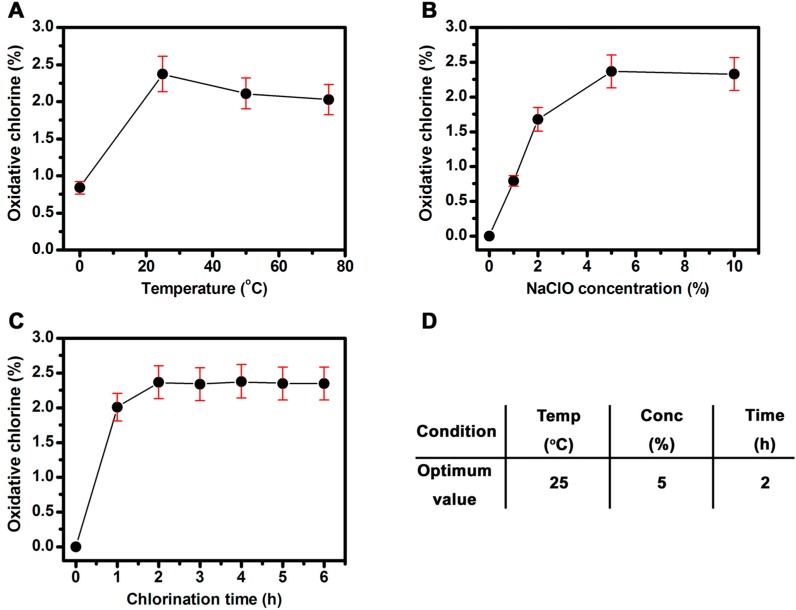
Effect of chlorination temperature (**A**), NaClO concentration (**B**), and chlorination time (**C**) on the oxidative chlorine content in SiO_2_@PS/*N*-halamine NPs. The optimal chlorination conditions for preparing SiO_2_@PS/*N*-halamine NPs (**D**).

Figure 3A shows the plot of the oxidative chlorine content *vs.* reaction temperature under the conditions of 5 wt % of NaClO concentration and a chlorination time of 2 h. A drastic increase of chlorine content is obtained with heating up from 0 °C to 25 °C, reflecting the increase in the *N*-halamine loading on the NPs before 25 °C. The beneficial effect on chlorine content is possibly due to the fact of the accelerated rate of chlorination induced by raising the system temperature. However, a gradual decrease in chlorine content is detected with further temperature increases from 25 °C to 50 °C, and then to 75 °C. Based on this result, we speculate that the higher temperature might, to some extent, lead to the decomposition of N-Cl bond, resulting in the decrease of chlorine content despite the accelerated rate of chlorination. Another reason could be that the rising temperature might accelerate the decomposition of NaClO, which is also an explanation for the decrease in chlorine content at higher temperature.

The bleaching concentration also plays a significant role in tuning the oxidative chlorine content. To ascertain this effect, the NaClO concentration was varied from 1 wt % to 10 wt % (Figure 3B), with the chlorination temperature and aging time fixed at 25 °C and 2 h, respectively. The chlorine content steeply increases when the NaClO concentration is from 1 wt % to 5 wt %, and the highest content is found at 5 wt %. The reaction rate decreases, and the chlorine content is almost saturated with further increases in the NaClO concentration. Accordingly, it can be concluded that a sodium hypochlorite concentration of 5 wt % is high enough for the chlorination treatment.

Figure 3C shows the effect of chlorination time on the oxidative chlorine content using 5 wt % NaClO at 25 °C. The rising trend is pronounced at first, and slows down gradually as the chlorination time is extended further. The NPs reaches as high as 2.37% oxidative chlorine content upon chlorination treatment for 2 h. However, chlorination times longer than 2 h do not increase the chlorine content. Such a tendency in the chlorination process might be caused by the saturation behavior, suggesting that the chlorination can almost reach saturation within 2 h without extending the chlorination period. The hydrophobicity of *N*-halamines with high oxidative chlorine loading is another possible explanation for this phenomenon [26]. The N-Cl group can render the SiO_2_@PS/*N*-halamine NPs surface more hydrophobic, resulting in poorer contact with the aqueous bleaching solution and thus less oxidative chlorine loading. Taking all these results into consideration, the SiO_2_@PS/*N*-halamine NPs prepared with 5 wt % NaClO concentration at 25 °C for 2 h are the optimum candidate with the highest chlorine content, as shown in Figure 3D.

### 2.3. Antimicrobial Assay

**Figure 4:**
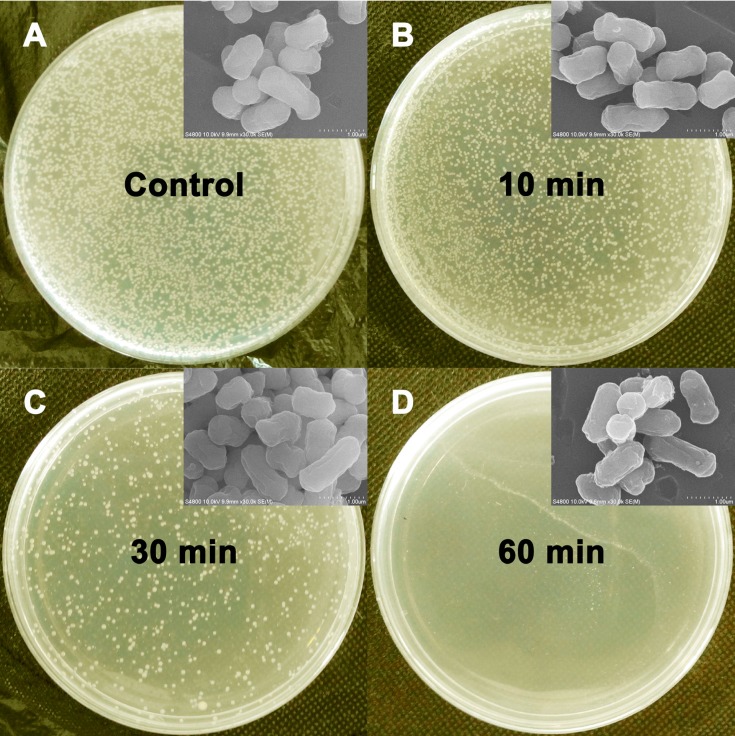
Photographs showing the bacterial culture plates of *E. coli* upon the exposure to the control (**A**) and SiO_2_@PS/*N*-halamine NPs (2.37% of the oxidative chlorine content) for different contact times (**B**–**D**). The insert shows SEM images of the corresponding *E. coli* treated with the control and SiO_2_@PS/*N*-halamine NPs for different contact periods.

The antibacterial activity of the SiO_2_@PS/*N*-halamine NPs was evaluated using *E. coli* as model bacterium via the modified plate counting method [27]. Figure 4 presents pictures of the LB culture plates, which reflect the bacterial survival upon extending the contact time with SiO_2_@PS/*N*-halamine NPs. Robust growth of *E. coli*, seen as dense small white dots, is found on the control plate (Figure 4A). Obvious reduction is detected in the population of the bacterial colonies after exposure to the SiO_2_@PS/*N*-halamine NPs for 10 min (Figure 4B). The reduction is more obvious when *E. coli* was treated with SiO_2_@PS/*N*-halamine NPs for 30 min (Figure 4C). No bacterial survival is seen on the culture plate after 60 min (Figure 4D), reflecting that the SiO_2_@PS/*N*-halamine NPs possess distinct antimicrobial capability for killing the model bacteria.

Morphological changes in *E. coli* after treatment were examined by SEM to further analyze the sterilization effects of the SiO_2_@PS/*N*-halamine NPs. The insert of Figure 4 shows the SEM images of *E. coli* before and after treatment with the SiO_2_@PS/*N*-halamine NPs for different contact periods. The intact *E. coli* is a typical corynebacterium with two obtuse ends and a smooth surface (insert of Figure 4A). After 10 min, rod-like shaped *E. coli* with a somewhat rugged surface are detected (Figure 4B). The roughness of bacterial surface tends to more obvious, and small holes even appear after 30 min (Figure 4C). Extending the contact time to 60 min, bacteria almost lose the original smooth appearance, and major crevasses (Figure 4D) is detected. More importantly, the SEM image presents cellular debris around the damaged outer membrane after 60 min. These results suggest that the SiO_2_@PS/*N*-halamine NPs can destroy the bacterial surface structures.

As for *N*-halamines, there are mainly two mechanisms for their antimicrobial activity, including the contact-killing mechanism and the release-killing mechanism [28]. From the plate counting method, we can speculate that the contact-killing mechanism is pausible explanation for the antibacterial action of SiO_2_@PS/*N*-halamine NPs. For a more in-depth study, a zone of inhibition study was also carried out. In this test, the control *E. coli* without antibacterial treatment present a robust growth showing crowded bacterial colonies without any inhibition regions, while *E. coli* treated with SiO_2_@PS/*N*-halamine NPs give an obvious inhibition ring. The release mechanism is a reasonable explanation for the existence of the inhibition ring. Based on the plate counting method and inhibition zone test, it might be concluded that SiO_2_@PS/*N*-halamine NPs kill bacteria by both the contact mechanism and the release mechanism.

### 2.4. Effect of Oxidative Chlorine Content on Antibacterial Activity

**Figure 5:**
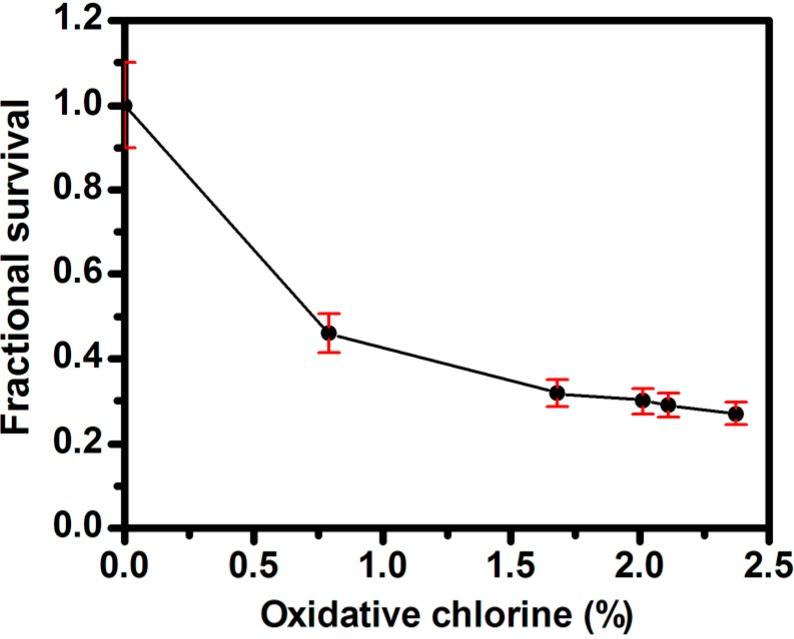
Effect of oxidative chlorine content of SiO_2_@PS/*N*-halamine NPs on the antibacterial activity.

As expected, the antibacterial capability of *N*-halamines is dependent on their oxidative chlorine content [29]. Herein, the effect of oxidative chlorine content on antibacterial capability was established by using *E. coli* as model bacterium. Figure 5 shows the fractional bacterial survival as a function of the oxidative chlorine loading after 30 min of treatment with SiO_2_@PS/*N*-halamine NPs. The fractional survival was calculated as the % survival = (*A*/*B*) (where *A* is the number of surviving bacterial colonies of the test sample and *B* is that of the control) [30]. As a general observation, the bacterial survival rate trend can be summarized as a “drastic increase first and then level off” mode, which is in good agreement with those described in other reports [31]. The most plausible reason is that despite the enhanced antibacterial efficacy with increasing chlorine content, the increase in oxidative chlorine loading also promotes the hydrophobicity of the NPs, which can prevent the products from contacting well with the bacteria. Thus the antibacterial performance is hindered, and as a result antibacterial efficacy decreases despite the increasing oxidative chlorine content.

### 2.5. Role of DksA Protein in Susceptibility of E. coli against N-halamine NPs

The antibacterial activity results fully verified the excellent biocidal activity of SiO_2_@PS/*N*-halamine NPs towards *E. coli*. In response to such a powerful antibiotic, the study regarding biological response of *E. coli*, *i.e.*, the susceptibility of *E. coli* towards the SiO_2_@PS/*N*-halamine NPs, was performed. It was reported previously that the DksA protein can play a significant role in determining susceptibility of *E. coli* [11]. Therefore, herein the sensibility of *E. coli* towards the SiO_2_@PS/*N*-halamine NPs induced by DksA protein was investigated.

**Figure 6:**
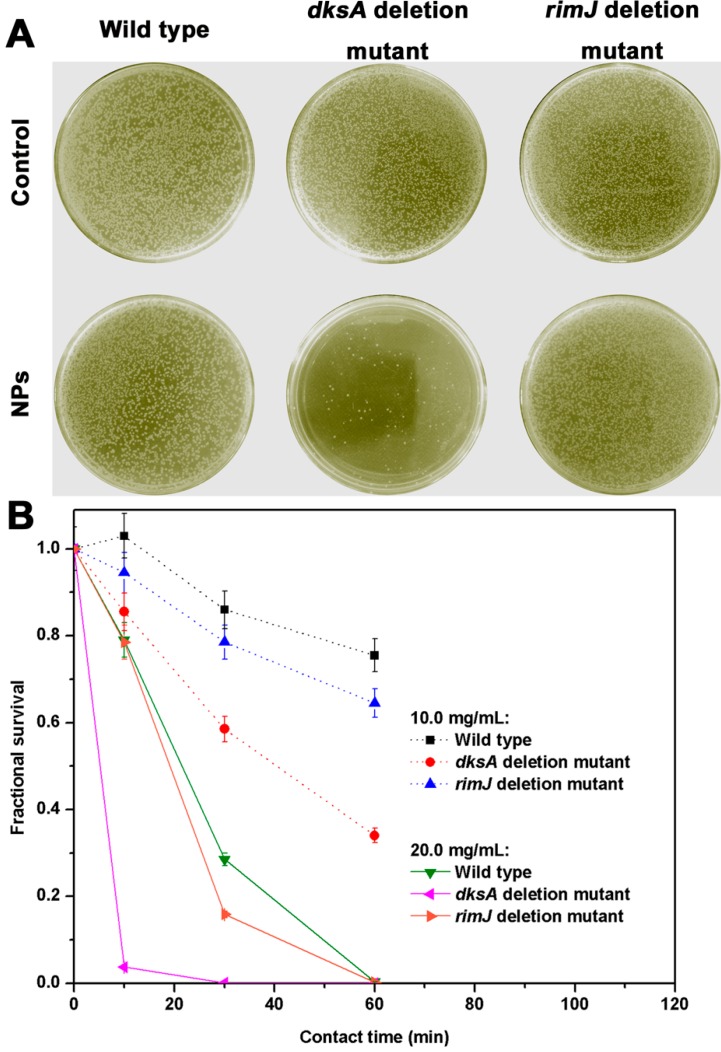
(**A**) Photographs showing the bacterial culture plates of the wildtype *E. coli*, *dksA* deletion mutant strain, and *rimJ* deletion mutant strain treated with the control and SiO_2_@PS/*N*-halamine NPs with concentration of 20.0 mg/mL for 10 min; (**B**) Time-kill assay curve of the wildtype *E. coli*, *dksA* deletion mutant strain, and *rimJ* deletion mutant strain treated with the SiO_2_@PS/*N*-halamine NPs with two different sample concentrations (10.0 mg/mL and 20.0 mg/mL).

To clarify the role of DksA, the *dksA* deletion mutant strain was constructed from an *E.* *coli* wildtype strain via replacing *dksA* with *cat* gene by a one-step inactivation method. The *rimJ* deletion mutant strain was also constructed by the similar way and used for comparison purposes. To determine whether DksA protein was involved in cell protection, a plate counting method was applied by treating the wildtype, *dksA* deletion mutant, and *rimJ* deletion mutant strain for 10 min with SiO_2_@PS/*N*-halamine NPs (20.0 mg/mL). The survivals of these three strains on an LB culture plate are shown in Figure 6A. Dense colonies are observed on the control plate for all three strains. The wildtype strain gives robust growth after NPs treatment, and the growth reduction is negligible. No remarkable difference is noticed either between the wildtype and *rimJ* deletion mutant strain, which means that the protein RimJ has almost no distinct effect on the susceptibility of *E. coli* towards the 20.0 mg/mL SiO_2_@PS/*N*-halamine NPs. In other words, the *rimJ* deletion mutant strain offers almost a similar resistance capability as the wildtype strain. This is the reason why we chose the *rimJ* deletion mutant strain as comparative control herein to corroborate the role of the DksA protein. A serious reduction in viability is detected in the population of the *dksA* deletion mutant colonies after the exposure to the SiO_2_@PS/*N*-halamine NPs. Only few colonies are seen on the culture plate, reflecting that the SiO_2_@PS/*N*-halamine NPs cause a great harm to the *dksA* deletion mutant strain. Such a result implies that the DksA protein is involved in cell protection against the SiO_2_@PS/*N*-halamine NPs. Our endeavor for designing this comparative assay is to identify whether the DksA protein is a decisive factor for determining the susceptibility of *E. coli* towards the *N*-halamines.

A time-kill assay is capable of detecting the rate and extent of antimicrobial activity, and thus is suitable for assessing changes in the antibacterial activity [32]. Herein, the time-kill assay was applied to further confirm the role of DksA protein in affecting the susceptibility of *E. coli* towards the SiO_2_@PS/*N*-halamine NPs. Figure 6B provides fractional survival rates of the wildtype, *dksA* deletion mutant, and *rimJ* deletion mutant strain challenged with 10 mg/mL and 20 mg/mL SiO_2_@PS/*N*-halamine NPs, respectively, as a function of contact time within the range from 0 to 60 min. Showing a similar tendency, the bacterial survival for all three curves shows faster reduction rates firstly and then a leveling off with the treatment duration time, which is in well agreement with the previous reports [33].

Interestingly, the *dksA* deletion mutant strain presents a remarkably sharp decrease of its fractional survival, while the wildtype and *rimJ* deletion mutant strain give gradual dropping trends with the aging time. At 10.0 mg/mL, the fractional survival of the *dksA* deletion mutant strain is only 30% after 60 min exposure, while the wildtype and the *rimJ* deletion mutant strain have as high as >70% survival rates. Therefore, we can conclude that the *dksA* deletion mutant strain is more sensitive than the wildtype and *rimJ* deletion mutant strains towards SiO_2_@PS/*N*-halamine NPs, which is much more obvious at the concentration of 20.0 mg/mL. The *dksA* deletion mutant strain is obviously completely dead after 30 min, while the wildtype and the *rimJ* deletion mutant strain reach 100% killing after 60 min. Accordingly, we can conclude that DksA is a significant protein in determining the susceptibility of *E. coli* to *N*-halamines.

The time-kill curve also shows a concentration-dependent activity of SiO_2_@PS/*N*-halamine NPs against *E. coli*. For all three types, the antibiotic effects change from a bacteriostatic to a bactericidal action with increasing sample concentration. Taking the *dksA* deletion mutant strain as an example, the bactericidal activity was fast-acting at 20.0 mg/mL concentration, and the bactericidal endpoint is only 30 min. Comparatively, the NPs with 10.0 mg/mL has not reached the bactericidal endpoint before 30 min, and still display about 35% survival even after extending the contact time to 60 min. Besides, we can also find a significant concentration-dependent relationship for the role of DksA protein from the time-kill test. As mentioned above, the determining effect of the DksA protein on the susceptibility of *E. coli* is well established at 10.0 mg/mL, and the differences in survival among these three strains after treatment with SiO_2_@PS/*N*-halamine NPs is further confirmed at 20.0 mg/mL. Thereby, we can speculate that the effect of the DksA protein on the susceptibility of *E. coli* towards the *N*-halamines is related to sample concentration, which is a decisive factor for their antibiotic action.

## 3. Experimental Section 

### 3.1. Materials

2,2,6,6-Tetramethyl-4-piperidinol (TMP) was obtained from Nangong Shenghua Chemicals Co., Ltd. (Nangong, China) Tetraethoxysilane (TEOS), styrene, and hydrochloric acid were obtained from the Tianjin Guangfu Fine Chemical Research Institute (Tianjin, China). 3-(Methacryloxy)propyl trimethoxy-silane (MPS) and azobisisobutyronitrile (AIBN) were available from Shanghai Chemical Reagent Plant (Shanghai, China). 1,4-bis(Chloromethyoxy)butane (BCMB) was purchased from Westingarea Co., Ltd. (Shanghai, China) Tin chloride pentahydrate, potassium hydroxide, toluene, dichloromethane, and sodium hypochlorite was provided from Sinopharm Chemical Reagent Co., Ltd. (Shanghai, China) The other reagents were analytical grade and were used without any purification.

### 3.2. Characterization

The morphology, particle size, surface state, and size distributions of the samples were observed on a SSX-550 field emission scanning electron microscope (SEM, Shimadzu, Kyoto, Japan) and a H8100 transmission electron microscope (TEM, Hitachi, Tokyo, Japan). The samples were dispersed in ethanol with assistance of sonication and cast onto a silicon wafer for SEM and copper grid for TEM characterization, and then dried at room temperature before examination. Dynamic light scattering (DLS) was measured by a ZetaPlus Zeta Potential Analyzer (ZZPA, Brookhaven, MS, USA). X-ray photoelectron spectra measurements were taken on a PHI-5000CESCA system (XPS, PHI, Chanhassen, MN, USA) with Mg K radiation (hr = 1253.6 eV). FTIR spectra were captured using a Nicolet Avatar 370 FTIR spectrometer (Thermo, Woburn, MA, USA) using the KBr pellet method in the range of 400–4000 cm^−1^ to analyze the sample compositions. The transmittance mode at a resolution of 4 cm^−1^ by averaging 32 scans was utilized. TGA was performed using a Perkin-Elmer thermogravimetric analyzer (Perkin-Elmer, Norwolk, CT, USA).

### 3.3. Preparation of MPS Modified SiO_2_ NPs

In detail, tetraethyl orthosilicate (TEOS, 25 mL) was added to a mixture of ethanol (40 mL), deionized water (50 mL) and ammonia (25 wt %, 30 mL). The mixture was stirred vigorously at room temperature for 12 h. Then 3-(methacryloyloxy)propyl trimethoxysilane (MPS, 2 mL) was added dropwise into the above mixture and stirred for 12 h. The as-prepared MPS modified SiO_2_ NPs (MPS-SiO_2_ NPs) were purified by several cycles of centrifugation and redispersion in a 1:1 (v:v) mixture of ethanol and water [34].

### 3.4. Preparation of SiO_2_@PS NPs

Typically, MPS-SiO_2_ NPs (0.5 g) and styrene (3 mL) were added to toluene (20 mL), and polymerization initiated by azoisobutyryldinitrile was performed under nitrogen atmosphere at 80 °C for 12 h to obtain SiO_2_@PS NPs. The as-synthesized NPs were centrifuged and washed several times to remove the impurities, and dried in a vacuum [35].

### 3.5. Immobilization of TMP onto SiO_2_@PS NPs

Immobilization of TMP on SiO_2_@PS NPs was accomplished via a two-step process including chloromethylation of SiO_2_@PS NPs and TMP immobilization [35]. Firstly, chloromethylation of SiO_2_@PS NPs was performed as follows. SiO_2_@PS NPs (0.5 g) was dispersed into dichloromethane (20 mL) and the chloromethylation reagent 1,4-bis(chloromethyoxy)butane (3.0 g) and SnCl_4_ catalyst (0.2 mol/L, 1.0 mL) were injected slowly into the mixture and stirred at room temperature for 24 h. After centrifugation, the chloromethyled SiO_2_@PS NPs were obtained. Subsequently, TMP (0.397 g) was mixed with freshly distilled anhydrous THF (50 mL). The mixture was stirred in a sealed flask under a nitrogen atmosphere at ambient temperature, and then poured into a flask containing NaH (0.066 g). The reactants were stirred for 30 min, after that chloromethyled SiO_2_@PS NPs (0.5 g) were added into the mixture. The reaction was continued for 12 h at 60 °C to obtain SiO_2_@PS/TMP NPs [36].

### 3.6. Synthesis of SiO_2_@PS/N-halamine NPs

Chlorination of SiO_2_@PS/TMP NPs was carried out as follows: SiO_2_@PS/TMP NPs (about 0.5 g) were dispersed into sodium hypochlorite solution buffered at pH 7 (10 wt %, 100 mL). The chlorination was carried out by vigorously stirring to obtain SiO_2_@PS/*N*-halamine NPs [37,38].

### 3.7. Determination of Oxidative Chlorine Content

Oxidative chlorine content in the SiO_2_@PS/*N*-halamine NPs was determined by the iodometric/thiosulfate titration procedure [39]. The percentage of oxidative chlorine (Cl %) was calculated according to the following equation:

$$Cl\text{\%}=\frac{35.5}{2}\;\times \;\frac{\left(V_{Cl}-V_{0}\right)\;\times 10^{-3}\times 0.01}{W_{Cl}}\;\times 100$$

where V_Cl_ and V_0_ are the volumes (mL) of sodium thiosulfate solutions consumed in the titration of the chlorinated and unchlorinated samples, respectively, and W_Cl_ is the weight of the chlorinated sample (g).

### 3.8. Antimicrobial Evaluation

*E.* *coli* (a Gram-negative bacterium) was used as model bacterium to test the antibacterial activities of the products. Bacteria were grown overnight at 37 °C under agitation (250 rpm) in Luria Bertani (LB) growth medium. Cells were harvested by centrifugation, washed twice with phosphate-buffered saline, and diluted to concentrations of 10^7^ CFU/mL [40]. The products (about 10 mg) were dispersed in sterilized distilled water (0.45 mL), vortexed, and then sonicated for 30 min. For antibacterial tests, bacterial suspension (50 µL) was added into sample suspension (450 µL), mixed well, and incubated under constant shaking. After a certain period of contact time, 0.03 wt % sodium thiosulfate aqueous solution (4.5 mL) was added into the reaction suspension to neutralize the active chlorine and stop the antibacterial action of the sample. The resulting mixture was mixed well, serially diluted, and then 100 µL of each dilution was dispersed onto LB agar plates. Colonies on the plates were counted after incubation at 37 °C for 24 h.

### 3.9. Role of DksA Protein in Susceptibility of E. coli

In this test, *Escherichia coli* wildtype and two mutants (*dksA* and *rimJ* deletion mutant strain) were used. Cells were grown at 37 °C and samples taken until optical density OD_600_ = 0.8 for LB medium with shaking at 180 rpm. 1 mL of bacterial liquid with 10^0^ was taken and diluted until 10^−3^. Then 10 μL of bacterial liquid from the 10^−3^ mixing was taken twice into two EP tubes, respectively, one for the experimental group while the other for control. Different concentration of the SiO_2_@PS/*N*-halamine NPs (90 μL) were added with good contact for 1 h, then Na_2_S_2_O_3_ solution (900 μL) was added to neutralize the active chlorine and stop the antibacterial action. The same procedure was carried out for the control test by replacing the SiO_2_@PS/*N*-halamine NPs with sterilized water. The mixture was spread onto LB plates for overnight culture, and cell sensitivity to the SiO_2_@PS/*N*-halamine NPs was detected by colony counting.

## 4. Conclusions

Novel *N*-halamine-containing antibacterial NPs, *i.e.*, SiO_2_@PS/*N*-halamine NPs, with a core-shell structure are fabricated by anchoring *N*-halamine groups on PS-modified SiO_2_ NPs to fight against pathogenic bacteria. The highest oxidative chlorine content of the SiO_2_@PS/*N*-halamine NPs (2.37%), which determined via iodometric/thiosulfate titration. These were obtained with 5 wt % NaClO concentration at 25 °C for 2 h. The plate counting method, inhibition zone study, and time-kill assay show that SiO_2_@PS/*N*-halamine NPs possess excellent antibacterial capability towards the model pathogenic bacterium *E. coli*. Antibacterial tests suggested that SiO_2_@PS/*N*-halamine NPs combine the contact-killing mechanism and the release-killing mechanism to kill bacteria. The effect of the oxidative chlorine content on antibacterial activity is also investigated. Finally, the role of DksA protein in the susceptibility of *E. coli* toward SiO_2_@PS/*N*-halamine NPs was proven. Systematic investigation of the SiO_2_@PS/*N*-halamine NPs provides novel ideas for developing *N*-halamine NPs as potent antibiotics for killing bacteria or even disease control.
